# Epiphysiodesen und Hemiepiphysiodesen: Wachstumsstopp und Wachstumslenkung an der unteren Extremität

**DOI:** 10.1007/s00132-022-04219-8

**Published:** 2022-03-31

**Authors:** Madeleine Willegger, Maryse Bouchard, Reinhard Windhager, Alexander Kolb, Catharina Chiari

**Affiliations:** 1 Universitätsklinik für Orthopädie und Unfallchirurgie, Klinische Abteilung für Orthopädie, Medizinische Universität Wien, Währinger Gürtel 18–20, 1090 Wien, Österreich; 2 Division of Orthopaedics, Hospital for Sick Children, Toronto, ON Kanada

**Keywords:** Wachstumslenkung, Deformitätenkorrektur, Beinlängendifferenz, Kind, Zuggurtungsplatte, Guided growth, Deformity correction, Leg length discrepancy, Child, Tension band plate

## Abstract

Durch das Prinzip der Wachstumsblockade mittels Epiphysiodese und der Wachstumslenkung durch Hemiepiphysiodese können sowohl Beinlängendifferenzen als auch sagittale, koronare und schräge Achsdeformitäten an der unteren Extremität während des Wachstums korrigiert werden. Es werden temporäre und permanente Techniken unterschieden. Der große Vorteil liegt in der minimal-invasiven Anwendung und den geringen Komplikationen. Essenziell sind die genaue Planung sowie das exakte Timing, besonders wenn permanente Verfahren angewandt werden. Die Anwendung rund um das kindliche Kniegelenk kann als Goldstandard der Behandlung von Beinlängendifferenzen und Varus- und Valguskorrekturen bezeichnet werden. Die Wachstumslenkung an der unteren Extremität hat über die letzten Jahre viele neue Einsatzmöglichkeiten an der Hüfte und am Sprunggelenk gefunden. Die erfolgreichen klinischen Ergebnisse mit geringen Komplikationen unterstützen die breite Anwendung der Hemiepiphysiodese und Epiphysiodese am wachsenden Skelett bei Achsfehlstellungen und Beinlängendifferenzen.

## Abstract (en)

The principals of growth arrest by epiphysiodesis and growth guidance by hemiepiphysiodesis are effective and powerful surgical techniques in pediatric orthopedics. These procedures can be used to correct leg length discrepancies as well as sagittal, coronal and oblique deformities. A differentiation is made between temporary and permanent techniques. The most significant advantage is that these techniques are minimally invasive and have low complication rates compared to acute osteotomy and gradual deformity correction. For optimal outcome an exact preoperative planning is needed to ensure accurate timing of the procedure, especially when permanent epiphysiodesis techniques are used. Although epiphysiodesis and hemiepiphysiodesis around the pediatric knee are most frequently used and can be considered the gold standard treatment of coronal plane deformities and leg length discrepancies, novel techniques for the hip and ankle are increasingly being performed. The successful clinical results with low complications support the broad use of hemiepiphysiodesis and epiphysiodesis for a variety of indications in the growing skeleton with deformities and leg length differences.

## Lernziele

Nach Lektüre dieses Beitrags …

- ist Ihnen das Prinzip der Epiphysiodese und Hemiepiphysiodese bekannt,
- sind Sie mit dem physiologischen Verlauf des Wachstums und der Beinachsen vertraut,
- kennen Sie die Grundlagen der Wachstumsprognose und des Timings der Epiphysiodese,
- wissen Sie, welche Techniken der Wachstumslenkung an der unteren Extremität (Hüfte, Knie, Sprunggelenk) Anwendung finden,
- sind Sie informiert über die klinischen und radiologischen Ergebnisse der Epiphysiodese und Hemiepiphysiodese rund um das kindliche Kniegelenk,
- sind Ihnen die potenziellen Komplikationen der Wachstumslenkung an der unteren Extremität (Hüfte, Knie, Sprunggelenk) bekannt.

## Einleitung

Der Begriff **Epiphysiodese**

Epiphysiodese

(von gr. „epiphyesthai“ – darauf wachsen und gr. „desis“ – binden) bedeutet, dass die Wachstumsfuge durch eine chirurgische Intervention verschlossen wird. Durch das Prinzip der Epiphysiodese (ED) können Beinlängendifferenzen (BLD) am wachsenden kindlichen Skelett ausgeglichen oder angeglichen werden. Essenziell ist eine genaue Planung, da ein zu früher Verschluss der Fugen zu einer Überkorrektur (zu kurze Extremität) oder ein zu später Verschluss zu einer Unterkorrektur (zu lange Extremität) führen kann. Eine klinische und radiologische Observanz der Achsverhältnisse im Verlauf ist ebenfalls wichtig, um ggf. sekundäre Deformitäten frühzeitig zu erkennen und zu behandeln [1, 2, 3, 4]. Eine **Hemiepiphysiodese**

Hemiepiphysiodese

(HED) betrifft nur eine Hälfte der Wachstumsfuge, wodurch ein weiteres Wachstum auf der nicht verschlossenen Seite gegeben ist. Dadurch können Achsfehlstellungen graduell über das Restwachstum auf der konkaven Seite der Deformität korrigiert werden. Mittels HED können je nach Platzierung und Lokalisation sowohl sagittale, koronare als auch schräge Achsdeformitäten an der unteren Extremität korrigiert werden. Die HED bei Deformitäten des heranwachsenden Kindes ist sehr effektiv und vermeidet die Komplikationen von akuten Korrekturen durch Osteotomien oder graduellen Korrekturen durch externe Fixierung (Fixateur externe) [5, 6, 7].

## Grundprinzipien

Hueter lieferte 1862 erstmals eine wissenschaftliche Erklärung für das Phänomen der mechanischen Beeinflussung des Knochenwachstums, als er berichtete, dass ein erhöhter Druck parallel zur Achse der Epiphyse das Wachstum hemmt, während ein verringerter Druck das Wachstum fördert [8]. Sieben Jahre später stellte Volkmann fest, dass Veränderungen der **Druckkräfte**

Druckkräfte

ein asymmetrisches Wachstum eines Gelenks verursachen. Diese Beobachtungen, die vor fast 150 Jahren gemacht wurden, legten den Grundstein für das Konzept der **Epiphysenklammern**

Epiphysenklammern

und haben andere Aspekte der pädiatrischen orthopädischen Praxis beeinflusst [9]. Die Beziehung zwischen Belastung und epiphysärer Modellierung ist jedoch komplexer, als es das Hueter-Volkmann-Gesetz vermuten lässt. Die Theorie der „**chondralen Modellierung**

chondralen Modellierung

**Figure 1:**
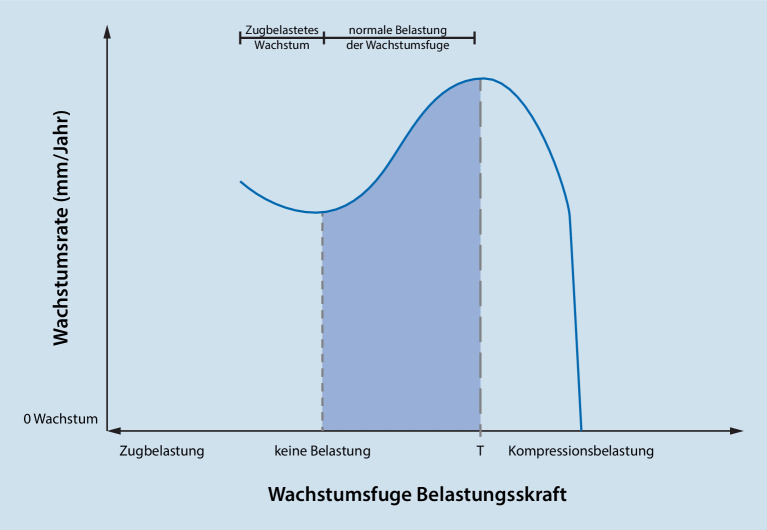

“ von Frost besagt, dass die Beziehung zwischen Belastung und chondralem Wachstum einer umgekehrten U‑Form ähnelt ([10]; Abb. 1).

Physiologische **Belastungen**

Belastungen

stimulieren das Wachstum, während Belastungen außerhalb dieses Bereichs, ob höher oder niedriger, das Wachstum hemmen. Geringfügige **Inkongruenzen**

Inkongruenzen

im Gelenk, bei denen die Belastungen innerhalb der physiologischen Grenzen bleiben, rufen also eine negative Rückkopplung hervor, um das Gelenk wieder zu normalisieren. Eine zunehmende Inkongruenz führt dazu, dass die Wachstumsfuge Belastungen ausgesetzt wird, die außerhalb des normalen physiologischen Bereichs liegen, und ruft einen positiven Rückkopplungsmechanismus hervor, der zu einer fortschreitenden Deformierung führt [5]. Diese komplexe, nichtlineare Beziehung hat viele Auswirkungen auf die Behandlung von Deformitäten einschließlich eines Zeitfensters, außerhalb dessen eine Manipulation der Wachstumsfuge fehlschlagen kann. Vor allem aber legt sie nahe, dass jeder Eingriff in einem frühen Stadium erfolgen sollte, in dem eine **negative Rückkopplungskorrektur**

negative Rückkopplungskorrektur

genutzt werden kann. Eine frühzeitige Wiederherstellung der mechanischen Achse ist wünschenswert, um dauerhafte Anomalien an den angrenzenden Gelenkflächen zu vermeiden, die andernfalls zu langfristiger Morbidität führen würden.

## Evolution

Die initial 1933 von Phemister beschriebene ED-Technik zum definitiven Verschluss der Wachstumsfugen rund um das kindliche Kniegelenk beinhaltet eine Osteotomie mit Extraktion eines Knochenblocks auf Höhe der Wachstumsfuge [13]. Der **Knochenblock**

Knochenblock

wird rotiert und wieder eingesetzt, um einen Verschluss der Fuge zu erzielen. Diese Technik ist wirksam, wurde jedoch über die Jahre aufgrund der Invasivität großteils von weniger invasiven Techniken abgelöst. Die **perkutane Drillepiphysiodese**

perkutane Drillepiphysiodese

nach Canale mit Kürettage der Wachstumsfuge ist eine beliebte weniger invasive definitive Lösung mit reproduzierbaren Ergebnissen, solange auf eine korrekte Durchführung geachtet wird [3].

Inspiriert von Tierexperimenten von Haas, entwickelten Blount und Clarke ein Klammerimplantat zur temporären Blockade der Wachstumsfuge. Die **Blount-Klammer**

Blount-Klammer

war das erste Implantat das breite Anwendung fand. Komplikationen mit Implantatbruch, Dislokation und Extrusion der Klammern führten zu einem Korrekturverlust mit hohen Revisionsraten. Ein sog. **Rebound-Phänomen**

Rebound-Phänomen

nach Entfernen einer Blount-Klammer-HED mit überschießendem Wachstum der blockierten Fuge und erneuter Deformität konnte bei bis zu 40 % der Kinder und Jugendlichen beobachtet werden. Vor allem junge Patienten mit einem hohen Restwachstumspotenzial neigten zum Rebound, weshalb eine Überkorrektur bei Mädchen unter 12 Jahren und Jungen unter 13 Jahren empfohlen wurde [2, 14].

### Cave

Die Blount-Klammer-Epiphysiodese/Hemiepiphysiodese war mit hohen Implantat-assoziierten Komplikationsraten sowie dem Rebound-Phänomen verbunden.

Zusammenfassend führten diese Eigenschaften zu einer sehr hohen Unvorhersehbarkeit der Methode. Zur Behandlung der Beinlängendifferenz zeigten die Blount-Klammern ebenfalls nur mäßig zufriedenstellende Ergebnisse mit einem Drittel an Revisionsepiphysiodesen [15]. Métaizeau propagierte die **gekreuzte Schraubentechnik**

gekreuzte Schraubentechnik,

auch PETS („percutaneous epiphysiodesis using transphyseal screws“) genannt [16]. Eine ED mit gekreuzten Schrauben ist sicherlich die minimal-invasivste Technik. Ob diese Technik tatsächlich eine temporäre Wachstumsblockade hervorruft, bleibt umstritten. Bei einer adäquaten präoperativen Planung inklusive Timing, sollte es jedoch ohnehin zu keiner Überkorrektur kommen, weshalb eine temporäre Lösung zur ED eigentlich obsolet ist. Anzumerken ist, dass die perkutane Entfernung der Schrauben Probleme bereiten kann. Die Ergebnisse zur Behandlung der BLD als auch zur Achskorrektur sind jedoch unumstritten erfolgreich [17, 18]. Peter M. Stevens revolutionierte im Jahr 2005 die Wachstumslenkung mit einem neuen Implantat. Dieses besteht aus einer kleinen 2‑Loch-Platte mit niedrigem Profil, die extraperiostal angebracht wird und mit zwei 4,5 mm kanülierten nicht-winkelstabilen Vollgewindeschrauben zur Blockade der Wachstumsfuge implantiert wird. Die Platte wirkt entsprechend dem **Zuggurtungsprinzip**

Zuggurtungsprinzip

und ist dementsprechend ein Zuggurtungsplatten(ZGP)-Konstrukt. Das Fulcrum (Drehpunkt) liegt außerhalb der Wachstumsfuge, und es erfolgt die Ausübung einer Zugspannung auf die Wachstumsfuge. Während eine Klammer einen starren Drehpunkt innerhalb der Wachstumsfuge bildet, liegt das Rotationszentrum des Implantats außerhalb der Wachstumsfuge, wodurch ein längerer Hebelarm für das Wachstum entsteht, der theoretisch eine schnellere Korrektur bei gleichbleibender Gesamtlänge des Knochens ermöglicht [19]. Die Fähigkeit der Schraube, sich zu bewegen, führt zu einer geringeren Druckübertragung über die Wachstumsfuge, wodurch das Risiko einer Fusion der Fuge verringert wird [20]. Andere Implantate, die auf ähnlichen Prinzipien beruhen, sind in der Zwischenzeit ebenfalls am Markt. Diese vielfache Verbesserung der Anpassungsfähigkeit des Implantates gegenüber der Blount-Klammer hat zu einem erneuten Interesse an der Wachstumslenkung und folglich zu einer raschen Ausweitung seiner Indikationen geführt.

## Planung und Timing

Eine Grundvoraussetzung für die erfolgreiche Planung einer ED oder HED ist das Wissen über den physiologischen Verlauf des **Wachstums**

**Figure 2:**
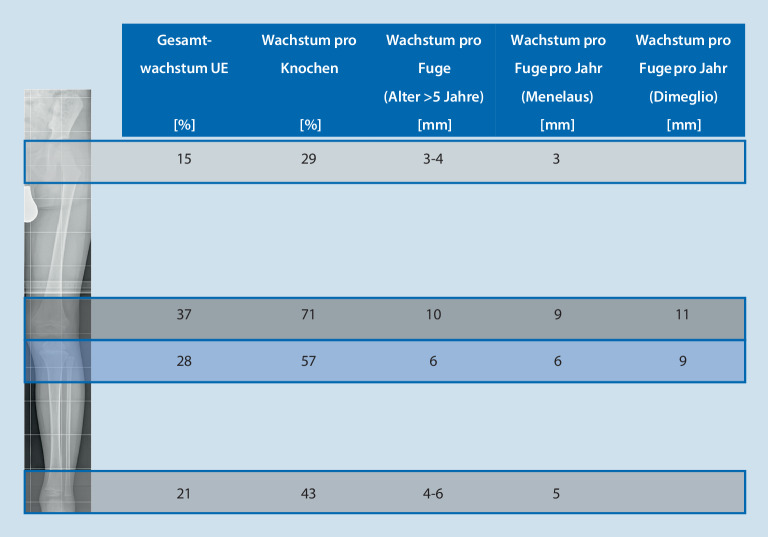

Wachstums

sowie über die normalen Achsverhältnisse an der unteren Extremität. Das Femur und die Tibia machen 54 % bzw. 46 % der Gesamtlänge der unteren Extremität bei der Skelettreife aus. Vier Wachstumsfugen (proximale und distale Femur- bzw. Tibiafuge) und der Fuß sind für das Längenwachstum verantwortlich, der größte Längenzuwachs findet um das Kniegelenk statt [21]. Anderson stellte fest, dass 71 % des Femurwachstums distal und 57 % des Tibiawachstums proximal stattfinden ([22]; Abb. 2). Die Gesamtwachstumsrate und das Wachstum der Extremitäten nehmen von der Geburt an ab, und zwar bis zur Pubertät, wenn der pubertäre Wachstumsschub einsetzt. Die unteren Gliedmaßen wachsen ab dem 5. Lebensjahr bis zur Pubertät im Durchschnitt 3,2 cm pro Jahr (ca. 2 cm pro Jahr am Oberschenkelknochen und 1,5 cm pro Jahr am Schienbein). Bis zum Beginn der Pubertät (Tanner-Stadium 2 und Skelettalter von 13 Jahren bei Jungen und 11 Jahren bei Mädchen) beträgt das verbleibende Wachstum der unteren Extremitäten im Durchschnitt 10 cm bei Jungen und 9 cm bei Mädchen, bevor die Skelettreife erreicht ist. Die Pubertät ist ein kurzer Zeitraum von etwa 2 Jahren mit raschen Wachstumsveränderungen. Die Wachstumsrate der unteren Extremitäten beschleunigt sich von 3,2 cm auf 5 cm pro Jahr am Höhepunkt der Pubertät [23, 24]. Das Alter zum Zeitpunkt des schnellsten Längenwachstums („peak height velocity“) liegt vor dem Einsetzen der Menstruation oder dem Auftreten des Risser-Zeichens bei Mädchen [25]. Menelaus definierte das Ende des Wachstums im Alter von 14 Jahren für Mädchen bzw. 16 Jahren für Jungen.

### Merke

Nach Menelaus ist das Ende des Längenwachstums bei Mädchen im Alter von 14 und bei Jungen im Alter von 16 Jahren erreicht.

Diese Formeln gelten nur, wenn die Pubertät im normalen Altersabschnitt beginnt. Das Wachstum eines Kindes mit Pubertas praecox oder tarda verhält sich anders, und eine enge interdisziplinäre Zusammenarbeit mit pädiatrischen Endokrinologen ist zu einer genaueren Analyse wünschenswert. Der beste Weg, das Wachstum einzuschätzen, sind möglichst exakte und **wiederholte Messungen**

wiederholte Messungen.

Je genauer und häufiger die Daten erhoben werden, desto sensibler und präziser ist die Wachstumsprognose, wodurch die chirurgische Therapie gezielter geplant werden kann. Strenge Analyse und Flexibilität bei der Interpretation sind der Schlüssel zum Erfolg.

### Achsverhältnisse an der unteren Extremität

**Figure 3:**
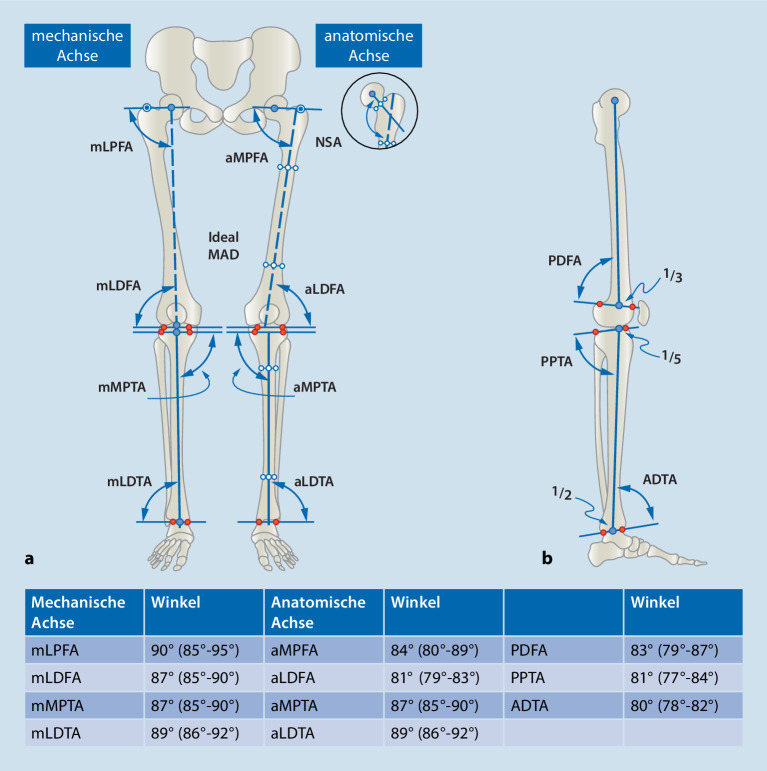

Es wurden Normwerte für die Ausrichtung der Beinachse in der Frontalebene festgelegt, die die mechanische Belastung der unteren Extremitäten beschreiben. Diese werden anhand eines stehenden Ganzbeinröntgens (anterior-posterior) vermessen. Es wird zwischen einer mechanischen und anatomischen Achse der korrespondierenden Knochen unterschieden. Für die Sagittalebene gibt es ebenfalls Normwerte, die anhand eines seitlichen Ganzbeinröntgens vermessen werden (Abb. 3).

Die **mechanische Achse**

mechanische Achse

der unteren Extremität ist die Linie, die den Mittelpunkt des Hüftkopfes mit dem Mittelpunkt des Sprunggelenks verbindet (Frontalebene). Wird die mechanische Achse relativ zur Mitte des Knies nach medial verschoben, entsteht ein Genu varum; wird die Achse umgekehrt relativ zur Mitte des Knies nach lateral verschoben, entsteht ein Genu valgum. Die sog. **„mechanical axis deviation“**

**Figure 4:**
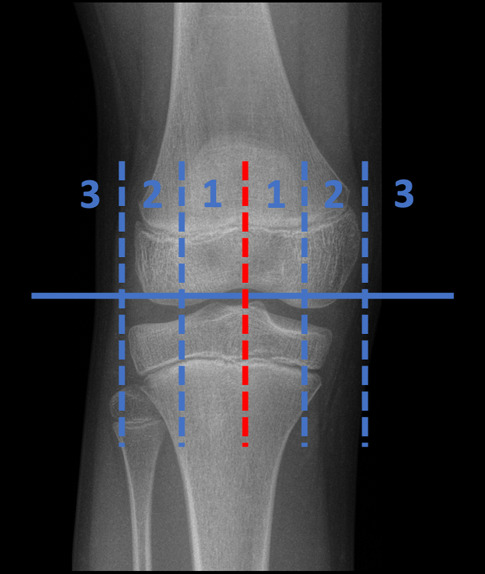

„mechanical axis deviation“

(MAD) kann in Millimetern vermessen werden [27]. Da die Normwerte der Achsverhältnisse am erwachsenen Skelett definiert wurden, sind für das kindliche Skelett andere Parameter ausschlaggebend. Der Winkel zwischen der mechanischen Achse des Femurs und der Tibia wird als **femorotibialer Winkel**

femorotibialer Winkel

bezeichnet. Die Entwicklung der Achsverhältnisse der unteren Extremitäten in der Frontalebene während der Kindheit wurde von Salenius und Vankka beschrieben [28]. Das normale Entwicklungsmuster des pädiatrischen femorotibialen Winkels ist symmetrisch und folgt einem einheitlichen Muster [29]. Neugeborene werden typischerweise in varus („O-Beine“) geboren. Im Alter von 18 bis 24 Monaten erreichen sie einen neutralen Winkel von 0°, anschließend entwickeln sie im Alter von etwa 3 Jahren einen Valguswinkel („X-Beine“). Salenius und Vankka definierten ein normales Alignement als einen endgültigen femorotibialen Winkel von 6° valgus, der im Alter von 7 Jahren erreicht wurde [11, 28]. Der femorotibiale Winkel ist jedoch ein indirektes Maß für die tatsächliche Ausrichtung der Beine. Er ermöglicht keine Analyse oder Quantifizierung des Verlaufs der mechanischen Kräfte, die beim Stehen auf das Knie wirken [26, 30]. Bei der Analyse von frontalen Achsabweichungen der Kniegelenke kann die MAD gemessen und nach der ursprünglich von Müller und Müller-Färber entwickelten Klassifikation in Zonen eingeteilt werden [31]. Diese Klassifizierung wird v. a. zur Indikationsstellung für wachstumslenkende Eingriffe im Kindesalter verwendet. Eine Achsabweichung, die in Zone 1 fällt, gilt als geringfügig und liegt wahrscheinlich im normalen Bereich. Liegt die MAD in Zone 2, ist die Fehlstellung bereits relativ schwerwiegend und erfordert eine Behandlung oder zumindest eine engmaschige Verlaufskontrolle, denn unbehandelt kann diese Fehlstellung rasch in Zone 3 wandern. Zone 3 ist eine absolute Indikation für einen wachstumslenkenden Eingriff ([11]; Abb. 4).

### Timing

Der wichtigste limitierende Faktor ist der adäquate Zeitpunkt der Epiphysiodese, das sog. „Timing“. Dies spielt v. a. beim Ausgleich von Beinlängendifferenzen eine essenzielle Rolle. Die Prognose der zu erwartenden Beinlängendifferenz bei abgeschlossener Skelettreife als auch der Zeitpunkt und die Lokalisation der Epiphysiodese (rund um das Kniegelenk) sind die Schlüsselfragen, die präoperativ zu beantworten sind.

#### Merke

Die Wachstumsprognose der definitiven Länge des Knochens oder Körpergröße ist mit einem gewissen Fehler behaftet, der bei der Operationsplanung und der Beurteilung des Operationsergebnisses berücksichtigt werden muss.

Auf der Grundlage der Daten von Anderson, Green und Messner wurden mehrere Methoden zur **Wachstumsvorhersage**

Wachstumsvorhersage

beschrieben und finden derzeit Anwendung [22]. Diese Daten umfassen die Femur- und Tibialängen von Kindern (Jungen und Mädchen) im Alter von 1 Jahr bis zur Skelettreife entsprechend dem chronologischen Alter. Für eine einfache Berechnung der zu erwartenden Beinlänge oder Beinlängendifferenz wird beispielsweise die aktuelle Länge des Femurs und der Tibia des „normalen“ Beins mit den Messungen von Anderson verglichen („Green-Anderson growth remaining chart“). Je nach Alter und Geschlecht kann die entsprechende Perzentile auf dem Diagramm definiert werden, und aus den Perzentilen kann die voraussichtliche Länge zum Zeitpunkt der Skelettreife abgelesen werden. Moseley hat diese Daten in eine Tabelle mit geraden Linien umgewandelt („Moseley straight line graph“), sodass die vorhergesagte Beinlänge aus dem Diagramm abgelesen werden kann, indem die aktuellen Beinlängen und das Skelettalter aufgetragen werden [32]. Menelaus und White beschrieben eine vereinfachte Methode zur Wachstumsprognose, die auf eigenen Berechnungen beruhte und besagte, dass die unteren Extremitäten pro Jahr um 23 mm wachsen und das größte Wachstum dabei aus den Wachstumsfugen rund um das Kniegelenk resultiert mit 15 mm (9 mm aus dem distalen Femur und 6 mm aus der proximalen Tibia) [33, 34]. Die derzeit populärste Methode ist die von Paley entwickelte **Multiplier-Methode**

Multiplier-Methode.

Auf der Grundlage der Daten von Anderson und Green sowie eines Datensatzes von Maresh [35], der radiologisch gemessene Längen von Femora und Tibiae von Säuglingen im Alter von 0 bis 1 Jahr enthält, wurde ein Multiplikator (M = „multiplier“) berechnet, der sich aus der erwarteten Länge des Knochens bei Skelettreife (Lm) geteilt durch die Länge zum Zeitpunkt des aktuellen Alters (L) ergibt: M = Lm / L. Das Restwachstum oder die erwartete BLD kann mit einer einfachen Formel berechnet werden [36].

Die **Dimeglio-Methode**

Dimeglio-Methode

ist ähnlich der arithmetischen Menelaus-White-Methode. Dimeglio berechnet das Wachstum am Knie mit 2 cm pro Jahr (1,1 cm am Femur und 0,9 cm an der Tibia) zu Beginn der Pubertät (Knochenalter von 11 Jahren bei Mädchen und 13 Jahren bei Jungen). In diesem Modell hört das Wachstum im Knochenalter von 13,5 Jahren bei Mädchen und 15,5 Jahren bei Jungen auf. Basierend auf dieser Grundlage, werden für jede Beinlängendifferenz ab 5 cm ein Zeitpunkt und eine Lokalisation der Epiphysiodese erstellt ([37]; Tab. 1).

**Table 1:** 

BLD	Lokalisation Epiphysiodese	Timing/Zeitpunkt
*5* *cm*	Femur + Tibia	Beginn Pubertät (Skelettalter: 13 Jahre Jungen, 11 Jahre Mädchen)
*4* *cm*	Femur + Tibia	Beginn Pubertät + 6 Monate
*3* *cm*	Femur	Beginn Pubertät
*2* *cm*	Femur	Beginn Pubertät + 1 Jahr
*2* *cm*	Tibia	Beginn Pubertät

Tabellen, Graphen und mittlerweile Smartphone-Apps können die Darstellung und Berechnung der Wachstumsprognose vereinfachen. Im Hinterkopf ist jedoch zu behalten, dass die Genauigkeit der Berechnung unter allen Methoden schlecht ist mit einer signifikanten Rate von Fehlprognosen zwischen 10 und 27 % mit einer Abweichung des endgültigen Wachstums von über 2 cm vom prognostizierten Wert [38]. Der behandelnde Kinderorthopäde sollte auch bedenken, dass einfache Berechnungsfehler in 18 % der Fälle nachweislich auftreten [39]. In einem direkten Vergleich der **Vorhersagegenauigkeit**

Vorhersagegenauigkeit

zeigte die ursprüngliche Green-Anderson-Methode für das verbleibende Wachstum die größte Korrelation zwischen der erwarteten und der endgültigen BLD nach Epiphysiodese. Dennoch generierten alle Methoden einen überkorrigierten Wert [40]. Durch die Anwendung mehrerer Methoden, Messwiederholungen im Laufe der Zeit und die Bestimmung des Skelettalters kann die Präzision der Prognose erhöht werden [24, 41]. Die Genauigkeit der Vorhersage der endgültigen Beinlängendifferenz nimmt zu, je älter das Kind wird (Kinder über 10 Jahre) [21].

#### Merke

Durch Messwiederholungen im Laufe der Zeit, die Verwendung des Skelettalters sowie die Berechnung mit mehreren Methoden können Fehlprognosen hinsichtlich des Restwachstums minimiert werden.

### Skelettalter

Das chronologische Alter basiert auf den tatsächlichen Lebensjahren. Das Skelettalter (Knochenalter) ist ein **Reifeindikator**

Reifeindikator,

der auf einer Reihe von röntgenologischen „Normen“ basiert, die uns Vorhersagen über das zukünftige Wachstum ermöglichen. Der Greulich-Pyle-Atlas beschreibt die Stadien der Verknöcherung auf einer einfachen dorsopalmaren (d/p) Röntgenaufnahme der linken Hand einschließlich des Handgelenks [42]. Die Tanner-Whitehouse- und die Short-Hand-Bone-Age-Methode sind weitere Verfahren zur Beurteilung des Knochenalters anhand einer Röntgenaufnahme der linken Hand [43, 44]. Die unterschiedlichen Merkmale der Verknöcherung im kindlichen Skelett können genutzt werden, um ein Knochenalter zu bestimmen und die Befunde mit dem chronologischen Alter des Kindes zu korrelieren. Eine Differenz von chronologischem Alter zum Skelettalter von ±1 Jahr oder mehr kann als akzeleriertes, normales oder retardiertes Knochenalter klassifiziert werden [45]. Während der 2 Jahre des pubertären Wachstumsschubs hat sich die **Sauvegrain-Methode**

Sauvegrain-Methode

als zuverlässig erwiesen. Die Sauvegrain-Methode bestimmt das Knochenalter mithilfe eines 27-Punkte-Scoring-Systems anhand einer anterioren/posterioren (a/p) und seitlichen Röntgenaufnahme des linken Ellbogens [46]. Der Ellbogen wird durch eine ausgeprägte Entwicklungsfolge seiner Ossifikationszentren definiert, die bei Mädchen im Alter von 9 und bei Jungen im Alter von 11 Jahren beginnt. Die Fusion der Wachstumsfugen des Ellenbogens ist bei Mädchen im Alter von 13 und bei Jungen im Alter von 15 Jahren abgeschlossen [43]; 50 % der Kinder haben ein akzeleriertes oder retardiertes Skelettalter [24]. Ein Knochenalter, das um mehr als 2 Standardabweichungen vom mittleren Alter abweicht, ist wahrscheinlich auf einen pathologischen Zustand zurückzuführen [47].

#### Merke

Die Verwendung des Skelettalters zur Berechnung der Wachstumsprognose erhöht die Genauigkeit der Prognose.

## Anwendungen und Ergebnisse rund um das Kniegelenk

### Epiphysiodesen rund um das Kniegelenk zur Beinlängendifferenzkorrektur

Im Allgemeinen ist die Epiphysiodese ein zuverlässiges und sicheres Verfahren zur Behandlung von BLD. Die ED wird am häufigsten am distalen Femur (dF), an der proximalen Tibia (pT) (mit oder ohne proximale Fibula [pF]) oder an beiden durchgeführt („Pan-genu“-Epiphyseodese).

**Komplikationen**

Komplikationen

im Zusammenhang mit der ED rund um das Kniegelenk werden zwar im Allgemeinen als selten angesehen, sind aber vielfältig. Dazu gehören Wundhämatome, postoperativer Hämarthros mit oder ohne begleitende Kniegelenksteifigkeit, oberflächliche oder tiefe Infektionen, vorübergehende oder dauerhafte Neuropathie, ungenaues Timing der Operation, das zu einer Unter- oder Überkorrektur der BLD führt, und ein unbeabsichtigter unvollständiger Epiphysiodeseeffekt, der eine Wiederholung der Operation erfordert oder die Entwicklung einer Achsdeformität mit sich bringt [16, 48, 49, 50]. Makarov et al. berichteten von einer Gesamtkomplikationsrate von 7 % bei 863 behandelten Kindern. In dieser Studie wurden die klassische Phemister-Technik, die offene Kürettage der Wachstumsfuge sowie die minimal-invasivere Drillepiphysiodese untersucht. Die Entwicklung einer Achsdeformität ist eine mögliche Komplikation, insbesondere bei kongenitaler BLD, jüngeren Patienten und größeren Längenunterschieden der Extremitäten [51].

Die perkutane **Drillepiphysiodese**

**Figure 5:**
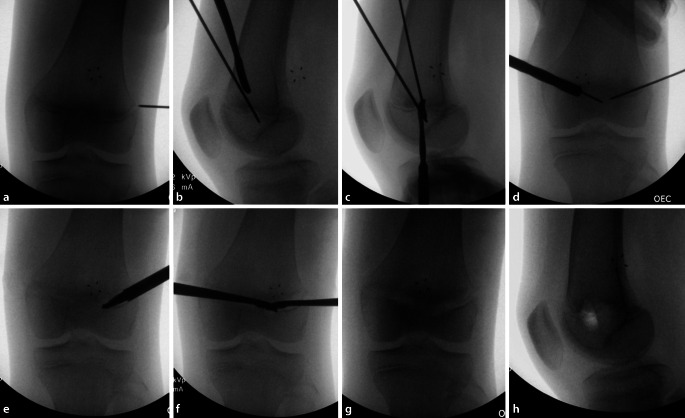

Drillepiphysiodese

stellt eine solide und wenig invasive Methode zur Behandlung von BLD dar. Sie wird meist durch 1 oder 2 Inzisionen von medial und lateral durchgeführt ([3]; Abb. 5). Eine zufriedenstellende Korrektur der BLD wurde in 82 % der Fälle berichtet, wobei eine asymmetrische Fusion oder Überkorrektur in 12 % der Fälle zu Komplikationen führte [52].

Die **PETS**

PETS

(„percutaneous epiphysiodesis using transphyseal screws“)-Technik mit einer gekreuzten Schraubenkonfiguration stellt eine minimal-invasive Methode zur ED dar. Vollgewindeschrauben aus Stahl mit einem großen Durchmesser (6,5 mm, 7,3 mm) werden verwendet, um die Wachstumsfuge zu blockieren. Es muss darauf geachtet werden, dass die Schrauben die Mitte der medialen und lateralen Fuge kreuzen. Eine Nachuntersuchung von 82 Patienten mit 126 behandelten Wachstumsfugen zeigten eine niedrige 3,7 %-Revisionsrate [53]. Wichtig ist, dass mindestens 4 Schraubengewinde in der Epiphyse platziert werden, um eine angemessene Verankerung zu erzielen [16, 54].

#### Merke

Bei der gekreuzten Schraubenepiphysiodese sollten mindestens 4 Gewindegänge die Epiphyse erreichen.

Die Verwendung von** Zuggurtungsplatten**

Zuggurtungsplatten

zur Epiphysiodese findet v. a. Anwendung bei jungen Kindern mit größer prognostizierten BLD, die zu jung für eine definitive ED rund um das Kniegelenk sind, um die Beinlänge Schritt für Schritt anzugleichen. Einerseits ist v. a. an der proximalen Tibia wenig Platz in der Epiphyse, um genug Schraubengewinde mit der PETS-Methode sicher zu platzieren, andererseits besteht dennoch die Gefahr eines definitiven Fugenverschlusses durch die gekreuzte Schraubentechnik. Fraglich ist, ob die Platzierung von medialen und lateralen ZGP an der proximalen Tibia einen sog. „roofing effect“ bewirkt und eine intraartikuläre Deformität kreiert [55]. Rezente Daten von Ballhause et al. zeigten bei 44 Patienten jedoch keine intraartikuläre Deformität [56].

Ob eine Epiphysiodese an der proximalen Fibula (pF ED) notwendig ist, wenn eine definitive ED der proximalen Tibia (pT ED) durchgeführt wird, um ein überschießendes Wachstum der Fibula mit proximaler Migration des Fibulaköpfchens zu vermeiden, bleibt umstritten. Es besteht wie bei jeder Operation im Bereich des Fibulaköpfchens die potenzielle Komplikation einer Verletzung des N. peroneus. Boyle et al. untersuchten retrospektiv 234 Patienten nach pT ED mit und ohne pF ED [57]. Innerhalb der Untergruppe der jüngeren Kinder (≥2 Jahre verbleibendes Wachstum zum Zeitpunkt der ED) gab es statistisch signifikante Unterschiede zwischen den Patienten mit und ohne pF ED zum Zeitpunkt der Skelettreife in Bezug auf den proximalen Tibia-Fibula-Abstand und das Verhältnis von Tibia zu Fibula („tibia:fibula ratio“). Bei 10 Patienten wurde ein offensichtliches **Überwachstum**

Überwachstum

der proximalen Fibula radiologisch bei Skelettreife festgestellt, darunter 5 von 55 (9 %) ohne pF ED und 5 von 179 (2,8 %) mit pF ED. In dieser Kohorte gab es keine Komplikationen durch die zusätzliche pF ED. Patienten mit relativem Überwachstum der proximalen Fibula waren alle symptomfrei und zeigten keine Irritation des N. peroneus [57]. Eine gleichzeitige pF ED scheint bei Patienten mit einer verbleibenden Wachstumszeit von 2 Jahren oder weniger nicht notwendig zu sein und verhindert nicht eindeutig ein zu starkes Wachstum des Fibulaköpfchens. McCarthy et al. beobachteten eine Wachstumsrate von 3 mm pro Jahr im Bereich der proximalen Fibula nach pT ED [49].

#### Merke

Eine proximale Fibulaepiphysiodese während der Durchführung einer proximalen Tibiaepiphysiodese ist potenziell bei Patienten mit einem verbleibenden Restwachstum von mehr als 2 Jahren anzudenken.

Im Fall eines **prognostizierten Hochwuchses**

prognostizierten Hochwuchses

wie beispielsweise beim familiären Hochwuchs, dem Marfan-Syndrom oder auch dem Beckwith-Wiedemann-Syndrom kann eine beidseitige Epiphysiodese der kniegelenknahen Wachstumsfugen durchgeführt werden. Die vorhandenen Studien zeigen eine Größenreduktion von ca. 5 cm. Im Allgemeinen gibt es keine Empfehlung für das perfekte Alter zur Korrektur eines zu erwartenden extremen Hochwuchses. Der Zeitpunkt sollte sich an den Wachstumsprognosen, der Pubertät und der gewünschten Größenreduktion orientieren [58].

### Epiphysiodese zur koronaren Achskorrektur (Genu varum, Genu valgum)

**Figure 6:**
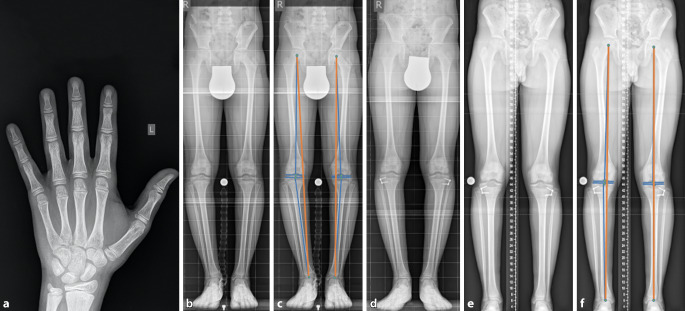

Genu varum und Genu valgum sind häufige Fehlstellungen des Kindes- und Jugendalters, ihre genaue Prävalenz ist jedoch unbekannt. Die unbekannte Prävalenz ist wahrscheinlich darauf zurückzuführen, dass die Deformität durch eine Vielzahl von Faktoren beeinflusst werden kann wie beispielsweise dem Geschlecht, der ethnischen Zugehörigkeit, der Ernährung, einer Krankheit und der körperlichen Aktivität [11]. Der Ursprung der **frontalen Achsabweichung**

frontalen Achsabweichung

kann auf das distale Femur oder die proximale Tibia zurückgeführt werden (s. Normwerte Abb. 3). In zahlreichen Reviews wurden die Studien zur Wirksamkeit von ZGP zur Korrektur von Deformitäten der koronaren Ebene im Bereich des Knies zusammengefasst ([5, 11, 59]; Abb. 6). Danino et al. untersuchten in einer Multicenterstudie die Korrektur mittels ZGP anhand von 206 Patienten (362 Wachstumsfugen). Sie erreichten in 93 % eine mechanische Achsausrichtung (mLDFA zwischen 85 und 89°) am Femur und in 92 % eine Winkelkorrektur der mechanischen Ausrichtung der Tibia (mMPTA zwischen 85 und 89°) [60]. Berichtete Komplikationen der Wachstumslenkung mit ZGP sind Schraubenmigration, Infektion, Über- oder Unterkorrektur, vorzeitiger Verschluss der Wachstumsfuge und gebrochene Schrauben/Platten. Das sog. Rebound Phänomen beschreibt den Rückfall in die Achsdeformität nach Entfernen der ZGP. Patienten mit pathologischen Wachstumsfugen, Cozen-Deformität (Valgusdeformität nach proximaler metaphysärer Tibiafraktur) und Patienten im Alter von <10 Jahren haben eine höhere Rebound-Rückfallquote [61, 62, 63].

#### Cave

Patienten mit pathologischen Wachstumsfugen (z. B. Rachitis, Blount etc.), Cozen-Deformität und junge Kinder mit einem Alter von unter 10 Jahren neigen zu einem Rebound.

Eine erhöhte **Rebound-Häufigkeit**

Rebound-Häufigkeit

wurde auch bei Patienten mit einer anfänglichen Deformität von >20° beschrieben [61]. Bei Patienten mit höherem Risiko empfiehlt Stevens [64] die Entfernung der metaphysären Schraube, sobald die gewünschte Korrektur erreicht ist, wobei die ZGP mit der epiphysären Schraube in situ belassen wird („**schlafende Platte**

schlafende Platte

“). Tritt ein Rebound auf, kann die metaphysäre Schraube wieder eingesetzt werden, ohne dass eine neue Platte eingesetzt werden muss. Die potenziellen Vorteile dieser Strategie müssen gegen die möglichen unerwünschten Auswirkungen ihrer Anwendung abgewogen werden. In einer kürzlich durchgeführten multizentrischen Studie, an der 3 Zentren in Argentinien und Chile beteiligt waren, wurden 28 Operationen mit „schlafenden Platten“ untersucht. Bei 22 % der Extremitäten musste die metaphysäre Schraube erneut eingebracht werden [65]. Es wurde auch eine **Überkorrektur**

Überkorrektur

vorgeschlagen, um den Rebound-Effekt abzuschwächen [66]. Da der Rebound-Effekt jedoch nicht vorhersehbar ist, kann eine deutliche Überkorrektur bei jedem mit Wachstumslenkung behandelten Patienten zu einer entgegengesetzten Deformität bei den Patienten führen, bei denen kein Rebound auftritt.

#### Cave

Ein Rebound-Effekt ist schwer vorhersehbar. Dementsprechend sind prophylaktische Überkorrekturen als riskant einzustufen.

### Epiphysiodese am anterioren distalen Femur

**Figure 7:**
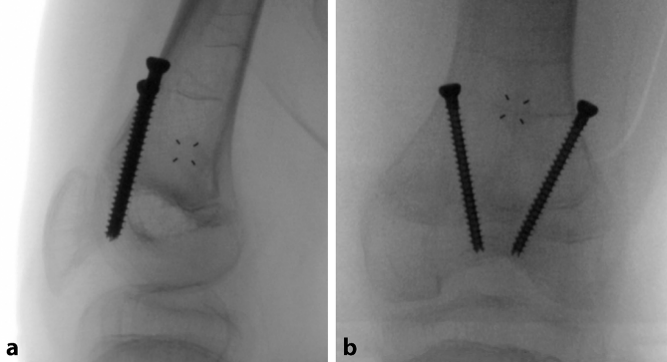

Patienten mit verschiedenen neuromuskulären Störungen, darunter Zerebralparese, Myelomeningozele, Arthrogrypose und andere Erkrankungen, weisen häufig **fixierte Kniebeugekontrakturen**

fixierte Kniebeugekontrakturen

auf. Bei einer fixierten Kniebeugekontraktur von mehr als 10° müssen chirurgische Optionen in Betracht gezogen werden, die jedoch meist sehr invasiv sind (ausgedehnte Weichteilrelease, Extensionsosteotomien des distalen Femurs). Es hat sich gezeigt, dass durch minimal-invasive Wachstumslenkung am distalen anterioren Femur Fehlstellungen in der Sagittalebene korrigiert werden können [67, 68]. Die Indikation sollte gestellt werden, solange noch ein Restwachstum von über 12 Monaten zu erwarten ist [6, 67]. Spiro et at. berichten, dass v. a. junge Kinder mit einer ausgeprägten Flexionsfehlstellung von mehr als 30° von der Wachstumslenkung profitieren [68]. Schrauben, Klammern oder ZGP können verwendet werden [6]. Eine Serie von 83 behandelten Kniegelenken zeigte eine Verbesserung der Flexionskontraktur von 21° (10–60°) auf 8° (0–50°) [69]. Patella alta kann eine Folgeerscheinung sein, die jedoch nicht als klinisch signifikant empfunden wurde [70]. Obwohl in der Literatur nur wenig darüber berichtet wird, werden die Platten oder Klammern nach einer anterioren HED am distalen Femur oft als störend empfunden [71]. Die Verwendung von fugenkreuzenden Schrauben stellt hier eine minimal-invasivere Methode dar (Abb. 7). Studiendaten mit einem Vergleich der Methoden sind nicht vorhanden. Schrauben können sich aufgrund der starken Wachstumsfuge jedoch verbiegen [6, 72].

## Anwendungen und Ergebnisse rund um das Sprunggelenk

### Epiphysiodese des medialen Malleolus

Eine **Valgusdeformität**

**Figure 8:**
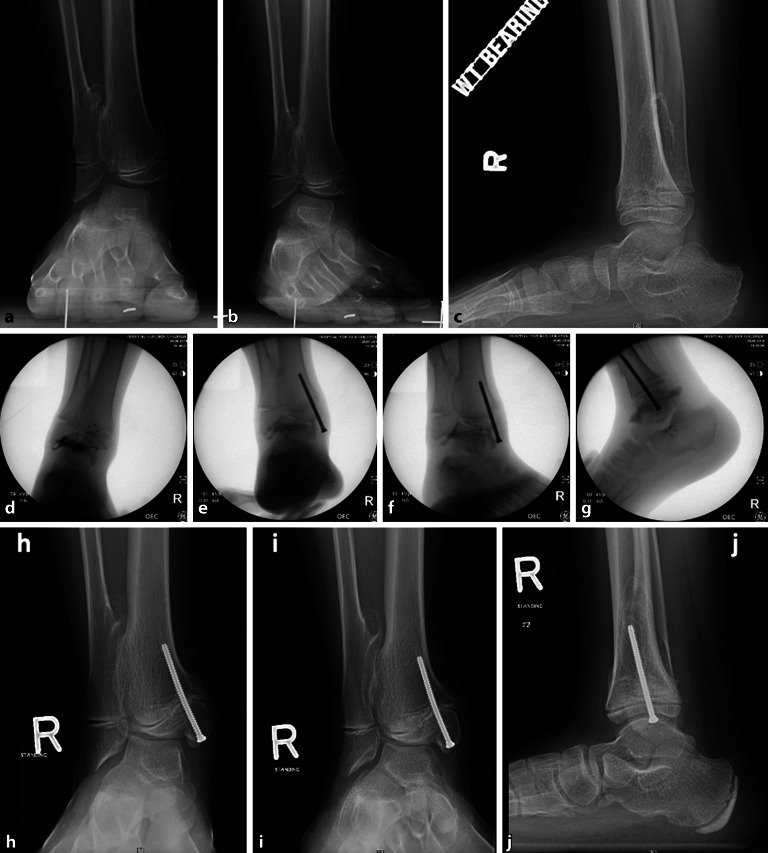

Valgusdeformität

des Sprunggelenks kann bei Kindern mit verschiedenen Erkrankungen auftreten. Diese Valgusfehlstellung wird mit der Zeit fortschreiten und evtl. zu einer Fehlbelastung und vorzeitiger Abnützung des Sprunggelenks führen [73]. Die Valgusdeformität wird am häufigsten bei Kindern mit neuromuskulären Störungen wie Myelomeningozele, Poliomyelitis und Zerebralparese beobachtet. Sie tritt auch bei Kindern mit postaxialer Hypoplasie, kongenitalem Klumpfuß, angeborener Pseudarthrose der Tibia oder hereditären multiplen Exostosen (HME) auf ([59]; Abb. 8).

Die Hemiepiphyseodese des medialen Malleolus (mM) mit Schrauben wurde erstmals 1997 von Stevens und Belle als einzelne vertikale Schraube beschrieben, die in der midkoronalen Ebene des Malleolus und die Wachstumsfuge kreuzend platziert wird, um einen Sprunggelenkvalgus zu korrigieren [74]. Die **HED des mM**

HED des mM

ist bei einem Valgus des Sprunggelenks von 5–8° bei Patienten mit offenen Wachstumsfugen indiziert. Als Implantat wird meist eine einzelne kanülierte 4,0- oder 4,5-mm-Vollgewindeschraube verwendet, und eine Korrektur von 9,7–12° wurde in klinischen Studien erzielt [73, 75]. Die korrekte Platzierung der **medialen Malleolusschraube**

medialen Malleolusschraube

(mMS) sowohl in der koronalen als auch in der sagittalen Ebene ist entscheidend. Je medialer (d. h. peripherer) die mMS in der koronalen Ebene platziert wird, desto stärker ist der Tethering-Effekt in Bezug auf die anschließende Winkelkorrektur, wie bei der partiellen Epiphysiodese und einem peripheren Physenverschluss nach einem Trauma zu beobachten ist [76]. Eine **intraoperative Durchleuchtung**

intraoperative Durchleuchtung

ist erforderlich, um zu vermeiden, dass die mMS zu weit lateral platziert wird, wodurch das Risiko eines Durchbruchs in das Sprunggelenk und einer Erosion der medialen Talusschulter besteht [6]. Eine sekundäre Deformität in der Sagittalebene kann auftreten, wenn die Schraube nicht zentral im medialen Malleolus platziert wird. Das Wiederauftreten der Valgusdeformität im Sinne eines Rebound-Effekts bei Kindern, bei denen die mMS vor der Skelettreife entfernt wurde, ist in vielen Studien beschrieben und deutet darauf hin, dass der Tethering-Effekt der Schraube reversibel ist. Stevens empfiehlt eine Überkorrektur von ca. 5° (varus), wenn die mMS noch bei offenen Fugen entfernt wird [6, 75, 76]. In keiner Arbeit wurde über einen Wachstumsstillstand nach Verwendung dieser Technik berichtet. Es wurden Bedenken hinsichtlich der Verwendung von mMS geäußert, da diese zu Infektionen führen können [74, 76]. Zuggurtungsplatten (ZGP) wurden ebenfalls erfolgreich zur Sprunggelenkvalguskorrektur eingesetzt [77]. Eine vergleichende retrospektive Studie zeigte, dass die **Korrekturgeschwindigkeit**

Korrekturgeschwindigkeit

mit der mMS um 50 % schneller ist als mit einer ZGP. **Chirurgische Komplikationen**

Chirurgische Komplikationen

wurden jedoch bei insgesamt 23 % der mMS-Knöchel im Vergleich zu 4 % der ZGP-Knöchel beobachtet. Zu den beschriebenen Komplikationen der mMS zählen die Schraubenmigration, die knöcherne Überwucherung des Schraubenkopfes mit erschwerter Materialentfernung, Schraubenbruch oder ein schmerzhafter prominenter Schraubenkopf. Zu den potenziellen Komplikationen der ZGP gehört die Infektion oder auch das Versagen des Konstrukts [78]. Kleine Epiphysen und osteopenischer Knochen mahnen zur Vorsicht bei der Verwendung von ZGP [79]. Generell wird die Wachstumslenkung zur Behandlung des Knöchelvalgus als sichere, gut tolerierte und effektive Operationsmethode angesehen [75].

### Epiphysiodese der distalen Tibia

**Figure 9:**
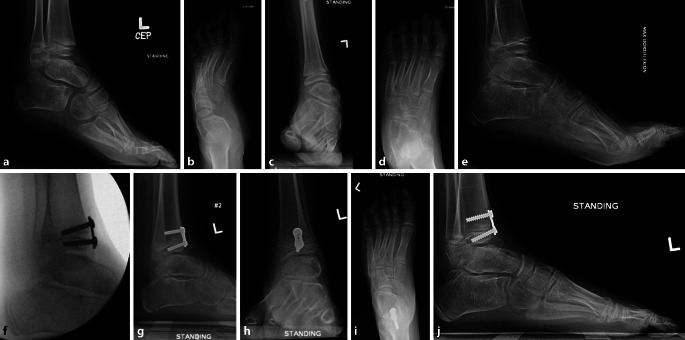

Die Hemiepiphysiodese der anterioren distalen Tibia wird zur Korrektur des **anterioren distalen Tibiawinkels**

anterioren distalen Tibiawinkels

(ADTA) v. a. bei residualen Spitzfußfehlstellungen nach Klumpfußbehandlung angewandt (Abb. 9). Ein erhöhter ADTA (>80°) in Kombination mit einem sog. Flat-top-Talus und einer eingeschränkten Dorsalextension des oberen Sprunggelenks stellt die Hauptindikation für dieses operative Verfahren dar. Die Verwendung von Klammern oder ZGP ist am gängigsten. Al-Aubaidi et al. berichteten über ihre Ergebnisse bei 31 Füßen und fanden eine mittlere Reduktion des ADTA von 13°. Die Verbesserung der Dorsalextension betrug im Mittel 2°, und es bestand keine Korrelation der Beweglichkeit mit der Winkelkorrektur. Die temporäre Hemiepiphysiodese wurde entfernt, wenn der gewünschte Effekt von etwa 15° Dorsalextension erreicht war oder der ADTA um mehr als 15° korrigiert wurde [80].

Eine neuartige Anwendung der Hemiepiphysiodese wurde von Laine et al. demonstriert, um Frakturen von Crawford-Typ-II- oder -III-Tibiae bei kongenitaler Tibiapseudarthrose („anterolateral tibial bowing/congenital tibial deficiency“) zu verhindern. Zehn Patienten wurden mittels anterolateraler distaler Tibiahemiepiphysiodese behandelt, und keiner entwickelte eine Tibiapseudarthrose oder eine Fraktur während des Follow-up von 5 Jahren [81].

## Anwendungen rund um das Hüftgelenk

### Epiphysiodese des Trochanter major

Um die mit dem übermäßigen Wachstum des Trochanter major (TM) verbundene Deformität zu verhindern oder zu minimieren, haben Chirurgen versucht, das Wachstum der TM-Apophyse zu verlangsamen oder zu stoppen. Im Jahr 1967 beschrieben Langenskiold und Selenius erstmals eine **Phemister-Hemiepiphyseodese**

Phemister-Hemiepiphyseodese

des lateralen TM bei Überwuchs im Rahmen einer avaskulären Nekrose der Hüfte [82]. Im Jahr 1980 untersuchten Gage und Cary 15 Patienten, bei denen derselbe Eingriff vorgenommen wurde. Sie berichteten über schlechtere Ergebnisse bei Kindern mit **Morbus Perthes**

**Figure 10:**
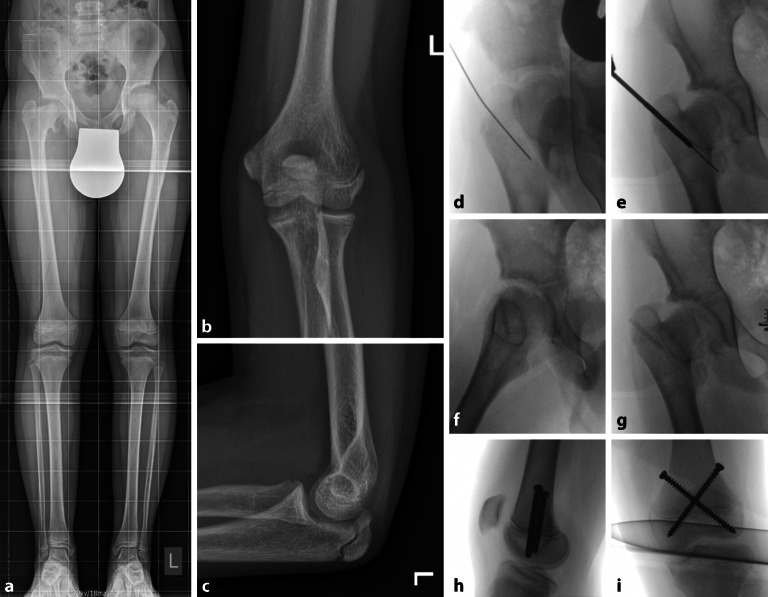

Morbus Perthes

als bei Kindern mit neonataler avaskulärer Nekrose oder Hüftdysplasie. Bessere Ergebnisse konnten erzielt werden, wenn die Operation vor dem 8. Lebensjahr durchgeführt wurde [83]. Das Alter zum Zeitpunkt der TM-Epiphysiodese dürfte eine große Rolle spielen. McCarthy und Weiner untersuchten 35 Kinder mit Morbus Perthes nach TM-Epiphysiodese, die mit einem Knochenstift, einer Drillepiphysiodese oder einer Schraubenepiphysiodese behandelt wurden. Das Wachstum des Trochanter major wurde um 0,9 mm/Jahr im Vergleich zur nichtbetroffenen Seite gehemmt. Eine stärkerer Effekt (1,8 mm/Jahr) wurde bei Kindern festgestellt, die mit einer Knochenstifttechnik operiert wurden, und überraschenderweise auch bei Kindern älter als 8 Jahre ([84]; Abb. 10). ZGP wurden auch am TM mit guten Ergebnissen bei Morbus Perthes verwendet [85].

### Epiphysiodese am Schenkelhals (proximale Femurwachstumsfuge)

Die Hemiepiphyseodese des inferomedialen Schenkelhalses wurde zur Korrektur der **Caput-valgum-Deformität**

Caput-valgum-Deformität

beschrieben, die durch einen lateralen Wachstumsstillstand (Typ II) der proximalen Femurwachstumsfuge im Rahmen einer Hüftdysplasie bedingt ist [86]. Eine große kanülierte Vollgewindeschraube (z. B. 7,3 mm) kann in den inferomedialen Aspekt der Femurfuge gesetzt werden, um so eine Wachstumslenkung mit mehr superolateralem Wachstum der Femurepiphyse zu kreieren. Torode und Young publizierten ihre Ergebnisse anhand von 11 Patienten (13 Hüften) mit lateralem Wachstumsstillstand nach medialer offener Reposition des Hüftgelenks. Signifikante Verbesserungen der femoralen und azetabulären Röntgenanatomie wurden nach der Hemiepiphyseodese festgestellt. In 5 Hüften war eine Revision der Schraube durch weiteres Eindrehen oder Auswechseln gegen eine längere Schraube notwendig [87].

Zur Behandlung der **Coxa valga**

Coxa valga

bei Patienten mit Zerebralparese besteht eine relativ gute Datenlage. Hsieh et al. behandelten 25 Kinder mit inferomedialer proximaler Femurhemiepiphysiodese aufgrund einer Coxa valga. Die Indikationen für eine Operation waren eine Subluxation des Femurkopfes mit einem Migrationsindex (MI) nach Reimers von >30 % und eine Coxa valga mit einem Centrum-Collum-Diaphysen(CCD)-Winkel von >155° bei einem verbleibenden Wachstum von mindestens 2 Jahren. Die Coxa-valga-Fehlstellung und die laterale Hüftsubluxation verbesserten sich mit einer Verkleinerung des CCD um durchschnittlich 13° und die Verringerung des MI um 10 %. Auch in diesem Patientenkollektiv war das „Auswachsen“ über die Schraubenlänge mit 43 % die häufigste Komplikation bzw. der häufigste Revisionsgrund [88]. Die **Schraubenposition**

Schraubenposition

dürfte eine Rolle spielen. Eine exzentrische (inferomediale) Position der Gewindegänge in der Epiphyse bewirkt einen biomechanisch effizienteren Hemiepiphysiodeseeffekt, hat jedoch den Nachteil einer kürzeren Länge des Gewindes in der kuppelförmigen Femurepiphyse und somit ein höheres Risiko des „Auswachsens“ [89]. Klinische Studien mit Langzeitergebnissen dieses Ansatzes sind erforderlich, um die Wirksamkeit der Wachstumslenkung bei der Korrektur von Deformitäten im Bereich des Hüftgelenks zu ermitteln. Der potenzielle Nutzen zur Minimierung invasiver Osteotomien ist nach wie vor ein wichtiger Impuls für diese Forschung.

## Fazit für die Praxis

- Die permanente Epiphysiodese (ED) rund um das Kniegelenk ist eine sichere und effektive Methode zur Behandlung von Beinlängendifferenzen, sofern das Timing mit einer adäquaten Wachstumsprognose korrekt ist.
- Die Wachstumslenkung durch Hemiepiphysiodese (HED) ist an der unteren Extremität eine etablierte Methode zur Deformitätenkorrektur.
- Die Anwendung der Zuggurtungsplatte hat die Wachstumslenkung durch eine verbesserte biomechanische Wirkung auf die Wachstumsfuge revolutioniert.
- Die Anwendung von Schrauben zur ED und HED bietet viele Optionen und gewinnt als minimal-invasive Technik immer mehr an Popularität.
- Komplikationen nach ED und HED sind selten und geringgradig.
- Das Überwachen des Wachstums nach Achs- oder Längenkorrektur durch ED und HED ist essenziell, um bei temporären Methoden eine rechtzeitige Materialentfernung zu indizieren und um potenzielle Achsabweichungen frühzeitig zu detektieren.
- Im Laufe der Zeit hat die Methode der Wachstumslenkung die invasiveren und aufwendigeren Osteotomien zunehmend verdrängt.

## CME-Fragebogen

In welcher Wachstumsfuge findet bei physiologischem Wachstum der Hauptteil des Längenwachstums statt?

Distale Tibiaepiphysenfuge

Epiphysenfuge des Hüftkopfes

Proximale Tibiaepiphysenfuge

Distale Femurepiphysenfuge

Trochanter Major

Ein 12-jähriger Junge wird wegen einer Beinlängendifferenz untersucht. Er hatte eine Osteomyelitis des linken distalen Femurs, die erfolgreich mit antibiotischer Therapie behandelt wurde. Ansonsten ist er gesund und hat keine weitere medizinische Vorgeschichte. Ganzbeinröntgenaufnahmen im Stehen bestätigen eine Beinlängendifferenz von 20 mm (2 cm), die auf einen Längenunterschied des rechten und linken Femurs zurückzuführen ist. Die Röntgenaufnahmen des Knies zeigen einen vollständigen Verschluss der linken distalen Femurwachstumsfuge ohne Achsdeformität. Wie groß ist die voraussichtliche Beinlängendifferenz im Erwachsenenalter, wenn sie unbehandelt bleibt? (Hilfestellung: Berechnung nach Menelaus)

2 cm

4 cm

6 cm

8 cm

10 cm

In welchem Alter kommt das Längenwachstum bei Jungen in der Regel an der Wachstumsfuge des distalen Oberschenkels zum Stillstand?

18

16

14

12

10

Ein 13-jähriger Junge stellt sich mit einer Beinlängendifferenz vor, wobei das rechte Bein kürzer ist als das linke. Er hat eine normale Größe für sein Alter, und sein Skelettalter entspricht seinem chronologischen Alter. Anamnese und Untersuchung nach Tanner zeigen, dass er vor 1 Monat in die Pubertät kam. Seine endgültige Beinlängendifferenz bei Skelettreife wird voraussichtlich 4,0 cm betragen. Welche der folgenden chirurgischen Optionen ist am besten geeignet?

Blount-Klammerepiphysiodese der medialen linken Tibia und Femurwachstumsfuge und Klammerentfernung mit 16 Jahren

Femurosteotomie und Verlängerung mittels Kallusdistraktion mit einem hexapoden Fixateur externe

Epiphysiodese des linken distalen Femurs in 1 Jahr nach Beginn der Pubertät

Epiphysiodese des linken distalen Femurs und der linken proximalen Tibia in 6 Monaten

Epiphysiodese beider distaler Femora und akute Verkürzungsosteotomie der linken Tibia (sofort)

Ein 14-jähriger Junge erleidet eine signifikante distale Femurfraktur (Salter-Harris Typ 2). Wie groß ist die prognostizierte Beinlängendiskrepanz, wenn man von einem vollständigen Wachstumsstillstand ausgeht?

Keine Beinlängendifferenz zu erwarten

1 cm

2 cm

3 cm

4 cm

Ein 8‑jähriges Mädchen wurde vor 2 Jahren wegen einer Salter-Harris-Fraktur Typ I des rechten distalen Femurs behandelt. Die Untersuchung zeigt eine symmetrische Knieflexion und -extension. Klinisch ist die Achse des Beines ident zur kontralateralen Seite (physiologisch). Sie hat jedoch eine Verkürzung des rechten Oberschenkels um 1 cm. Im Ganzbeinröntgen bestätigt sich eine Verkürzung des rechten Femurs um 1 cm. Aus der Anamnese geht hervor, dass sie immer in der 50. Perzentile der Körpergröße lag, und ihr Skelettalter entspricht ihrem chronologischen Alter. Was ist die zu erwartende Folge im Erwachsenenalter?

7 cm Beinlängendifferenz mit einem längeren rechten Femur

7 cm Beinlängendifferenz mit einem längeren linken Femur

12° Varusdeformität rechtes Bein

18° Valgusdeformität linkes Bein

20° Recurvatumdeformität linkes Bein

Bei einem 14-jährigen männlichen Patienten mit Beinlängendiskrepanz wird eine distale femorale und proximale tibiale Epiphyseodese am längeren Bein durchgeführt. Welches Ausmaß an Korrektur wird mit diesem Verfahren bei diesem Kind voraussichtlich erreicht? (Hilfestellung: Berechnung nach Menelaus)

1,6 cm

2 cm

3 cm

4 cm

6,4 cm

Ein 10-jähriges Mädchen stellt sich mit einer mehrjährigen Vorgeschichte von beidseitigen Knieschmerzen und einer Deformierung der unteren Extremitäten vor, wobei ihre Knie beim Laufen aneinander reiben. Es sind keine Erkrankungen bekannt. Es ist kein Trauma in der Anamnese. Ein Ganzbeinröntgenbild im Stehen ist in Abb. 11 dargestellt. Um welche typische Fehlstellung im Kindes- und Jugendalter handelt es sich?

Iatrogenes Genu recurvatum beidseits

Posttraumatisches Genu recurvatum beidseits

Posttraumatische Beinlängendifferenz

Idiopathisches Genu varum beidseits

Idiopathisches Genu valgum beidseits

Die einseitige Blockade der Wachstumsfuge (Hemiepiphysiodese) mittels Klammerung (Blount-Klammer) ist eine etablierte Methode zur Beinachsenkorrektur. Inwiefern ist die Klammer ein Beispiel für das Hueter-Volkmann-Prinzip?

Erhöhte Kompression entlang der Wachstumsfuge verlangsamt das Längenwachstum.

Verminderte Kompression entlang der Wachstumsfuge verlangsamt das Längenwachstum.

Erhöhte Spannung entlang der Wachstumsfuge verlangsamt das Längenwachstum.

Verminderte Spannung entlang der Wachstumsfuge verlangsamt das Längenwachstum.

Erhöhte Kompression entlang der Wachstumsfuge verstärkt das Längenwachstum.

Sie lesen das Konsil eines pädiatrischen Kollegen, der eine Überweisung zur Genu-valgum-Korrektur ausgestellt hat. Dieser beschreibt und eine MAD von 9 mm. Was bedeutet diese Abkürzung?

„Mechanical axis deviation“

„Multiple axis deviation“

„Multiple angle deformity“

„Multiple angle deviation“

„Magnitude angle deformity“

## Abkürzungen

ADTA: Anteriorer distaler Tibiawinkel
a/p: Anterior/posterior
BLD: Beinlängendifferenz
CCD: Centrum-Collum-Diaphysen-Winkel
dF: Distales Femur
d/p: Dorsopalmar
ED: Epiphysiodese
HED: Hemiepiphysiodese
HME: Hereditäre multiple Exostosen
IVO: Intertrochantäre Varisationsosteotomie
LDTA: Lateraler distaler Tibiawinkel
MAD: Mechanical axis deviation
mLDFA: Mechanischer lateraler distaler Femurwinkel
MI: Migrationsindex
mM: Medialer Malleolus
mMPTA: Mechanischer medialer proximaler Tibiawinkel
mMS: Mediale Malleolusschraube
PETS: Percutaneous epiphysiodesis using transphyseal screws
pF: Proximale Fibula
pT: Proximale Tibia
TM: Trochanter major
UE: Untere Extremitäten
ZGP: Zuggurtungsplatte
